# Harmonizing mouse anatomy terminology: a common language?

**DOI:** 10.1007/s00335-025-10156-6

**Published:** 2025-09-10

**Authors:** Jesús Ruberte, Paul N. Schofield, John P. Sundberg, Sergi Olvera-Maneu, Ana Carretero

**Affiliations:** 1 Department of Animal Health and Anatomy, Center for Animal Biotechnology and Gene Therapy, Universitat Autònoma de Barcelona, Travessera Dels Turons, 08193 Cerdanyola del Vallès, Barcelona, Spain; 2 Department of Physiology, Development and Neuroscience, University of Cambridge, Cambridge, UK; 3 The Jackson Laboratory, Bar Harbor, ME USA; 4 Department of Veterinary Medicine, University of Nicosia School of Veterinary Medicine, Nicosia, Cyprus

## Abstract

The mouse remains the principal animal model for investigating human diseases due, among other reasons, to its anatomical similarities to humans. Despite its widespread use, the assumption that mouse anatomy is a fully established field with standardized and universally accepted terminology is misleading. Many phenotypic anatomical annotations do not refer to the authority or origin of the terminology used, while others inappropriately adopt outdated or human-centric nomenclature. This inconsistency is further exacerbated by the limited availability of comprehensive anatomical references, often compelling researchers to rely on “do-it-yourself” anatomical interpretations when characterizing disease models—an approach that increases the risk of inaccuracies in the absence of expert anatomical guidance. To address this critical gap, we propose the formation of expert working groups comprising comparative anatomists and disease model developers. These groups would be responsible for systematically reviewing the anatomical literature of each mouse organ system and producing consensus-based terminologies aligned with the *Nomina Anatomica Veterinaria* (NAV), the authoritative standard for quadrupedal species. Such harmonization efforts would not only improve the consistency and reliability of anatomical descriptions in mouse models but also enhance the integration and interoperability of anatomical data across biomedical ontologies and databases, facilitating more robust data mining and translational research.

## Introduction

In recent decades, far-reaching developments in gene expression analysis, imaging, and the technology of mouse genome manipulation have significantly increased the importance of comparative mouse-to-human anatomy in basic and translational research (Gurumurthy et al. 2019; Clark et al. 2020; Pera et al. 2024).

It is often assumed that mouse anatomy is a well-established field, with a universally accepted terminology. After all, anatomy serves as the cornerstone of biomedicine, providing the necessary framework for understanding and describing mouse models of human diseases. However, this assumption is far from accurate. In fact, significant discrepancies persist, revealing a surprising lack of consensus.

Even more intriguingly, we now face a challenge strikingly similar to the one encountered during the nineteenth century in human anatomy, where anatomical nomenclature was chaotic and incoherent full of inequalities, contradictions, and obscurities (O’Rahilly 1989). If the human anatomical terms from various standard textbooks at that time were compiled into a single list, the total would exceed 30,000 terms. By comparison, the current Terminologia Anatomica (TA) (F.I.P.A.T. 2019) contains 7,635 terms. For instance, approximately fifty different terms existed for the epiphysis cerebri (O’Rahilly 1989).

**Fig. 1:**
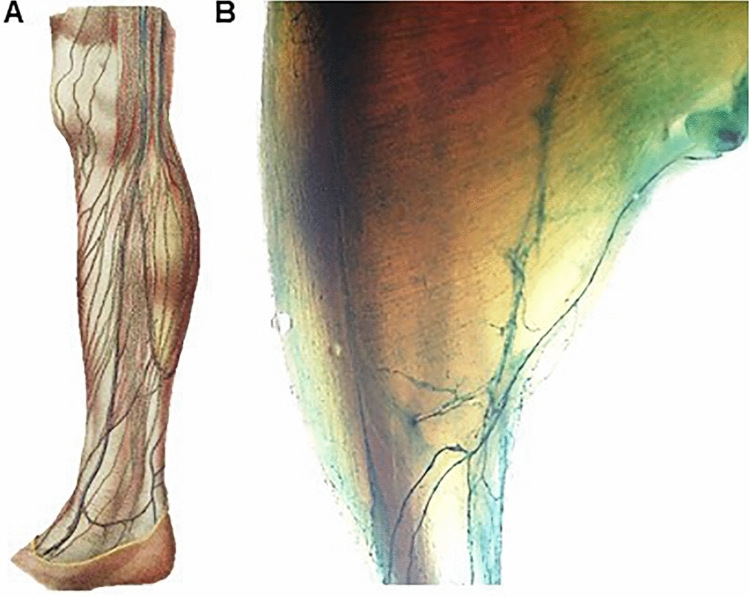
**A** Leg human lymphatic vessels (grey) in “Atlas d’Anatomie Descriptive du Corps Humain' (1844–1866) by Constantin Bonamy, Paul Broca and Emile Beau (Biblioteca Digital del Museo del Prado, Madrid, Spain). **B** Leg mouse lymphatic vessels (green) in a *Flt4*^***tm2.1(cre/ERT2)Sgo***^* kcnokin mouse*

There is no objective reason to expect that mouse anatomy should be less complex than human anatomy. The de facto gold standard for mouse nomenclature, Nomina Anatomica Veterinaria (NAV) (I.C.V.G.A.N. 2017), contains 6,545 terms, but the implementation of a widely-used nomenclature for phenotype and gene expression annotation, the Adult Mouse Anatomy Ontology (MA) (version 2017-02-07) contains approximately half of the terms present in the NAV (Hayamizu et al. 2005). Our target is, in addition, constantly moving as knock-in mice carrying anatomically-restricted gene expression markers are demonstrating anatomical details that were previously unknown. For example, acquisition of knowledge about the distribution of lymphatic vessels in humans had required centuries of meticulous injections of different colored compounds in extremely fragile and difficult to visualize vessels. However, with current models such as the *Flt4*^***tm2.1(cre/ERT2)Sgo***^ mouse (Martinez-Corral et al. 2016), it is now possible to directly and easily visualize the distribution of lymphatic vessels, showing a complexity comparable to that seen in humans (Fig. 1).

In this commentary we will consider several inconsistencies encountered in the current mouse gross anatomical terminologies used in books, articles, and databases. As will be shown, most of the resources do not refer to the authority or origin of the terminology used. Others use human terminology (a bipedal species) to describe mouse (a quadrupedal species), and in some of these resources, terminology is used inconsistently for both human and mouse. Furthermore, in several cases eponyms and other historical terms are used to name anatomical structures; eponymic use has long since been eliminated from formal human and veterinarian anatomical nomenclatures. Nevertheless, it is also relevant to note that insufficient knowledge of mouse anatomy leads researchers to “do-it-yourself” anatomy to describe and understand their models, which without the assistance of expert anatomists is prone to errors.

## Inconsistent use of anatomical terminology in the mouse: two illustrative cases

### Liver terminology

The absence of a gallbladder in rats and its presence in mice have led to mice being considered more similar to humans and a preferred model for hepatobiliary diseases. However, the human liver differs markedly from the mouse liver due to its architecture and a non-lobated external surface (Kruepunga et al. 2019). It has been hypothesized that in species with a very mobile vertebral column, such as mice, in comparison with species with a more rigid spine, hepatic lobes could more easily glide over each other, when the vertebral column is maximally flexed or extended (König and Liebich 2014).

Several mouse liver disease models, including inflammation and fibrosis (Iredale 2007; Fransén-Pettersson et al. 2016), alcoholism (Lamas-Paz et al. 2018), portal hypertension (Königshofer et al. 2022), and partial hepatectomy (Wang et al. 2022) have been developed during the last decades. For all of them, and especially for hepatectomy models, widely used to study liver tumor exeresis, liver failure, and liver regeneration (Wang et al. 2022), the exact anatomical identification of the liver lobes is essential.

**Fig. 2:**
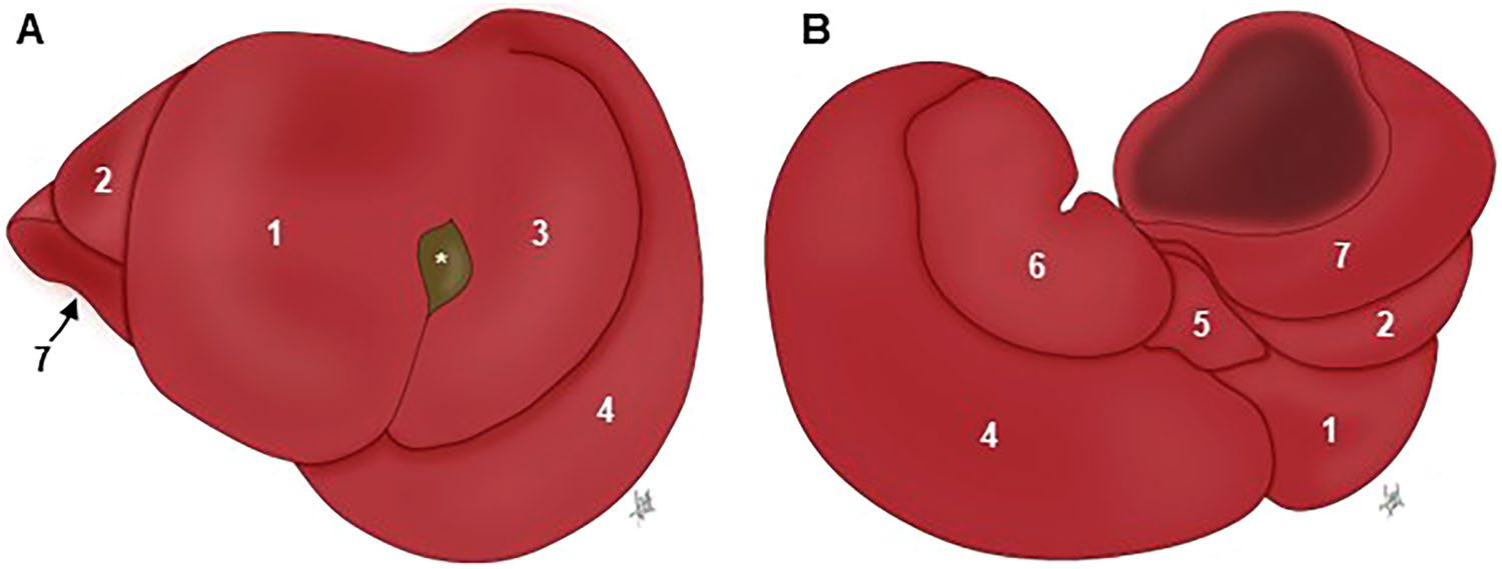
Mouse hepatic lobation following the NAV nomenclature. Captions are taken from an “in situ” fixed specimen. **A** Cranial view (diaphragmatic surface). **B** Caudal view (visceral surface). 1: Right medial lobe; 2: Right lateral lobe; 3: Left medial lobe; 4. Left lateral lobe; 5: Quadrate lobe; 6: Papillary process (caudate lobe); 7; Caudate process (caudate lobe); *: Gallbladder

Following NAV, the standard reference for anatomical terminology in quadrupedal species, the mouse liver has four lobes and four sublobes, as well as two processes. Both the left and right hepatic lobes are subdivided into a medial and lateral lobe, and the caudate lobe is subdivided into the caudate and the papillary process (Fig. 2). The quadrate lobe is found between the gallbladder and the round ligament, a postnatal remnant of the umbilical vein. The term “lobe” is used here as a portion of the liver demarcated by fissures.

Searching through bibliography and databases, we found thirteen books, eleven articles, and three databases describing the lobes of the mouse liver (Table 1). Three different groups of nomenclatures were used. Almost half of references (8 books, 4 articles, and 1 database) do not follow the NAV nomenclature describing a median lobe, generally corresponding to the right and left medial lobes of the NAV. In one of these groups, the median lobe is termed as “middle” lobe (MA ontology). Only three books (Popesko et al. 1992; Constantinescu 2011; Ruberte et al. 2017) and three articles (Fiebig et al. 2012; Nguyen et al. 2019; Kruepunga et al. 2019) followed the NAV nomenclature. The rest of the references partially followed the NAV, but miss the description of some of the lobes and/or named them differently (Table 1).

**Table 1:** Bibliographic analysis of terms used to name the liver lobes

Median lobe	1,6,10,13,16,17,20,22,23,24	**Quadrate lobe**	5,11,14,18,21,25,26,27,28
Left median lobe	10.15	**Caudate lobe**	1,3,4,5,6,7,10,11,12,13,14,16,17,18,19,20,21,22,23,24,25,26,27,28
Left portion of the median lobe	2,3,4,6	Superior caudate lobe	15
Right median lobe	10.15	Inferior caudate lobe	15
Right portion median lobe	2,3,4,6	Anterior caudate lobe	10
Middle lobe	26	Posterior caudate lobe	10
**Left lobe**	1,3,6,13,14,20,21,22,26,27,28	Dorsal portion caudate lobe	2,4,6
**Left medial lobe**	5,7,9,11,12,14,18,19,21,23,25,27	Ventral portion caudate lobe	2,4,6
Left upper lobe	8	Caudal lobe	2.7
**Left lateral lobe**	2,4,5,7,9,10,11,12,14,15,16,17,18,19,21,23,24,25,27,28	**Papillary process**	5,9,11,12,14,17,18,19,21,23,25,26,27,28
Left lower lobe	8	Omental lobe	8
**Right lobe**	1,3,9,10,13,14,16,17,20,21,22,24,26,27,28	Mamillary process	7
**Right medial lobe**	5,7,11,12,14,18,19,21,23,25	**Caudate process**	9,10,11,14,17,18,19,21,23,25,26
Right middle lobe	8		
Superior right lobe	10.15		
Right upper lobe	8		
Anterior sector of right lobe	28		
**Right lateral lobe**	2,4,5,6,7,11,12,14,18,19,21,23,25		
Right lower lobe	8		
Inferior right lobe	10.15		
Anterior portion right lateral lobe	2,4,6		
Posterior portion right lateral lobe	2,4,6		
Posterior sector of right lobe	28		
Upper sublobe	23		
Lower sublobe	23		

Terms in bold correspond to the NAV nomenclature

1. Cook (1965); 2. Hummel et al (1966); 3. Cook (1983); 4. Hollander et al (1987); 5. Popesko et al (1992); 6. Harada et al. (1999); 7. Iwaki et al (2001); 8. Greene and Puder (2003); 9. Cozzi et al (2006) in Italian; 10. Martins and Neuhaus (2007); 11. Constantinescu (2011); 12. Komárek (2012); 13. Rogers and Dintzis (2018); 14. Feibig et al. (2012); 15. Sänger et al (2015); 16. Nevzorova et al. (2015); 17. Higashiyama et al (2016, 2018); 18. Ruberte et al (2017); 19. Nguyen et al (2019); 20. Rogers and Dintzis (2018); 21. Kruepunga et al (2019); 22. Jena and Chawla (2021); 23. Dagli et al. (2022); 24. Wang et al (2022); 25. IMAIOS; 26. MA; 27. EMAPA; 28. UBERON

A special commentary is required for the quadrate and caudate lobes. It is generally assumed that the quadrate lobe is virtually absent in mouse (Kruepunga et al. 2019). However, all mouse databases surveyed, three books, and two articles refer to the existence of this lobe in the mouse (Table 1). Specifically, Popesko et al (1992), Constantinescu (2011), and Ruberte et al (2017) present respectively two diagrams and a photograph showing the shape and location of this lobe. On the other hand, the caudate lobe is practically described in all analyzed resources. However, only in three books, 4 articles, and 2 databases is the caudate lobe referenced along with its two processes, the papillary and the caudate (Table 1). Interestingly, Greene and Puder (2003) name the papillary process as an “omental lobe”. This terminology probably comes from considering the papillary process as the omental tuberosity in humans and carnivores, a projection on the visceral surface of left liver lobe. Similarly, Iwaki et al (2001) name the papillary process as the “mamillary” process, although we have not found a clear origin for this terminology. The Edinburgh Mouse Atlas Project (EMAPA) and Uber-anatomy (UBERON) ontologies include the term “bare area” in the left and right lobes to name the surface of the liver with no peritoneal covering. This term as “*area nuda*” is also included in the NAV and in TA. Finally, Cozzi et al (2006) describe two papillary processes.

Most of the resources surveyed do not acknowledge the origin of terminology used. Only, three books (Popesko et al. 1992; Constantinescu 2011; Ruberte et al. 2017) and two articles (Fiebig et al. 2012; Nguyen et al. 2019) cite NAV as the source of terminology. Iwaki et al (2001) follow the terminology of the Japanese Association of Veterinary Anatomist, which is a Japanese translation of NAV. In certain cases, the Latin NAV terminology was used (Popesko et al. 1992; Komárek 2012; Fiebig et al. 2012). Furthermore, one book and three articles used the terms “upper-lower” and “superior-inferior” belonging to the TA used for human anatomy (Greene and Puder 2003; Martins and Neuhaus 2007; Sänger et al. 2015; Dagli et al. 2022).

**Fig. 3:**
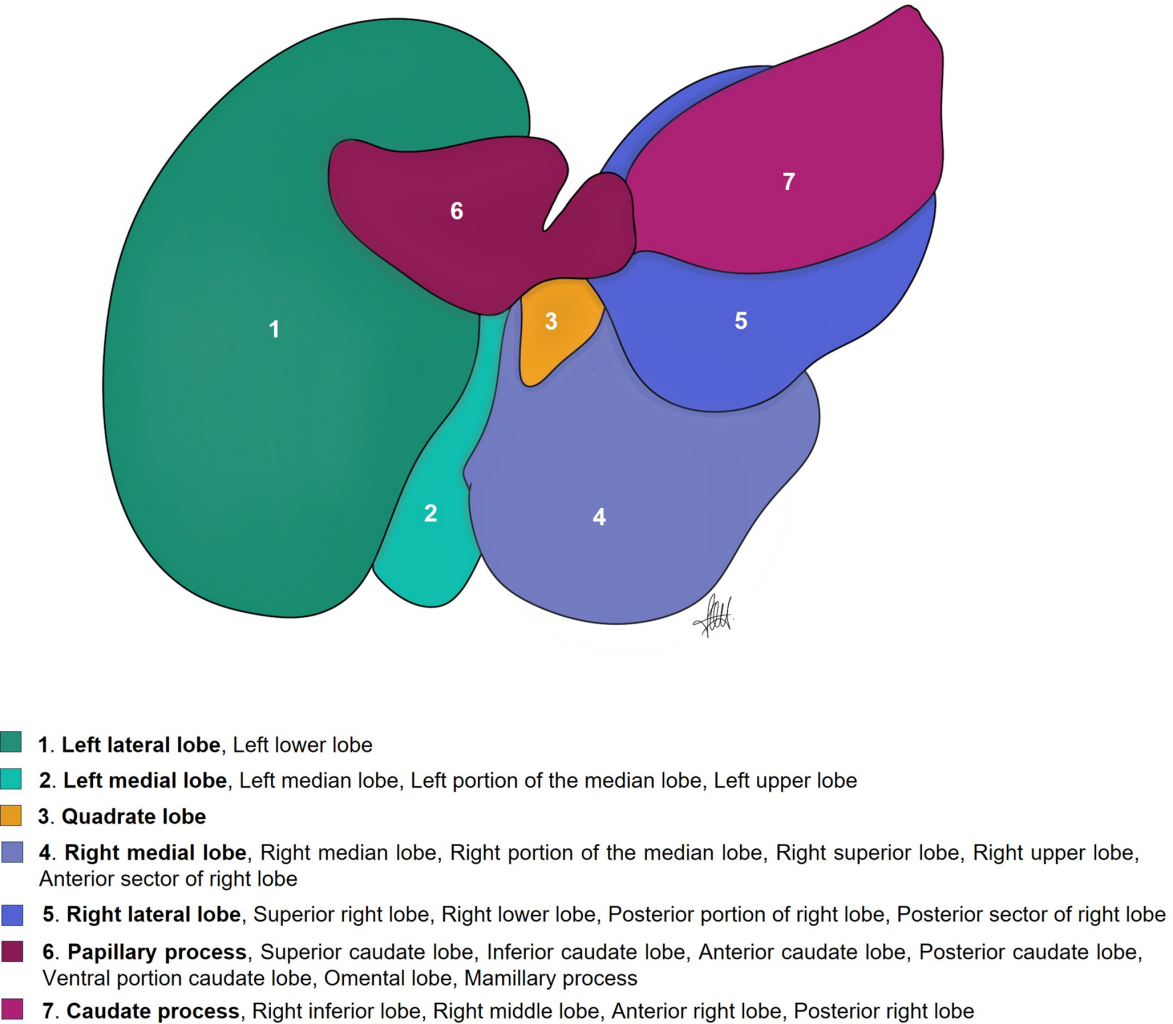
Different terms used to identify the liver lobes. The diagram represents the visceral surface of the liver isolated from the cadaver. Terms in bold correspond to the NAV nomenclature

In summary, of the eight terms accepted by NAV to describe the hepatic lobation, we have found thirty-six terms in the current assessed bibliography to name the mouse hepatic lobes, that is, twenty-eight more than those included in the NAV (Table 1). Furthermore, the use of diagrams and photographs in twenty-one of the analyzed resources allowed us to compare the identification made of the liver lobes through them and confirm the general lack of concordance (Fig. 3). As an example, the papillary process of the caudate lobe was identified just as caudate lobe, or by seven different terms (superior caudate lobe, inferior caudate lobe, anterior caudate lobe, posterior caudate lobe, omental lobe, mamillary process, and ventral portion caudate lobe).

One might think that the terminology applied to the lobation of the mouse liver is not crucial because has no physiological or clinical relevance, since in the non-lobated human liver, segments represented the physiological divisions used to define vascularization and hepatic resections. Couinaud and Nogueira (1958) divided the human liver by using the portal vein (subhepatic segmentation) and the hepatic veins (suprahepatic segmentation), splitting the liver in different blocks of liver tissue (segments) for resection. It is generally accepted that rodent hepatic lobes show equivalences to the human liver segments. In the rat the left lobe is equivalent to segment II; the middle lobe to segments III, IV, V, and VIII; and the right lobe to segments VI and VII (Kogure et al. 1999). In a more recent study describing the intrahepatic vascular anatomy in mouse (Sänger et al. 2015), it was also shown that lobar borders generally match vascular territorial borders, but not always due to vascular variability. Furthermore, inter-lobe differences in the murine hepatic innate immune response to endotoxemia have been noticed (Nguyen et al. 2019), pointing out the importance of a consensus in the terminology used and in the correct identification of liver lobes in mouse.

There are additional computational advantages of harmonising the terminologies to allow the assertion of homology in important cross-species ontologies such as UBERON (Mungall et al. 2012). The human anatomy ontology, FMA, lists the “Posterior segment of the right lobe of the liver” (FMA_14499), but its equivalent is not found in EMAPA or MA where it would be the “Right lateral lobe” or its synonyms (see Fig. 3), so harmonising the mouse and human ontologies would, for example, permit the listing of the structure and its homolog in two species, improving coverage and granularity. Interestingly “Posterior sector of the right lobe of the liver” is already in UBERON (UBERON_8600015) but with no cross-references bringing together the mouse and human regions.

### Forelimb muscle terminology

**Fig. 4:**
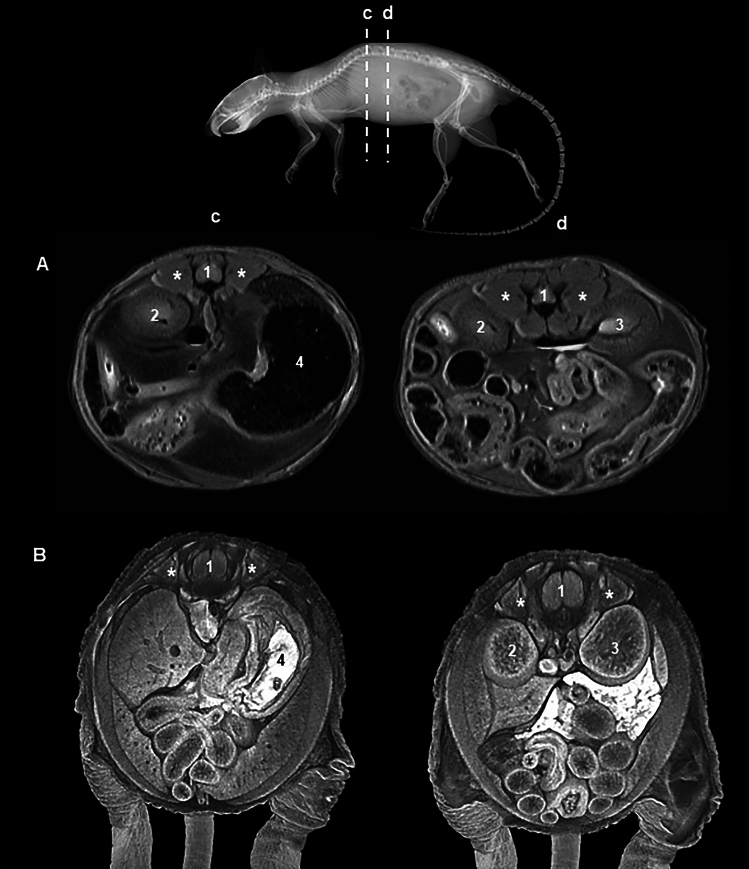
MRI (**A**) and iodine enhanced CT (**B**) transverse sections of mouse trunk. Note the epaxial muscles (*) around the spinal cord (1) enclosed in the vertebral canal. At these levels it should be possible to identify the transversospinal, interspinal, intertransverse, longissimus and iliocostal muscles, however, both resolution and current anatomical knowledge make their identification difficult. 2: Right kidney; 3: Left kidney; 4: Stomach

The knowledge about the skeletal gross muscle anatomy in mice is incomplete and fragmentary, probably due to the difficulty in dissecting and isolating their small and thin muscles. Specific mouse models like the Scleraxis (Scx)-GFP reporter (*Scx*^***tm1.1(EGFP)Skom***^) (DeLaurier et al. 2008) have been developed to visualize muscle tendons and preclinical imaging technologies, such as MRI, ultrasonography, and µCT using enhancers, allowing the visualization of mouse muscles (Zhang et al. 2008; Jeffery et al. 2011; Mele et al. 2023). However, the lack of knowledge boundaries, origins, and insertions in many muscles makes it largely difficult to identify them, especially in the trunk (Fig. 4).

**Fig. 5:**
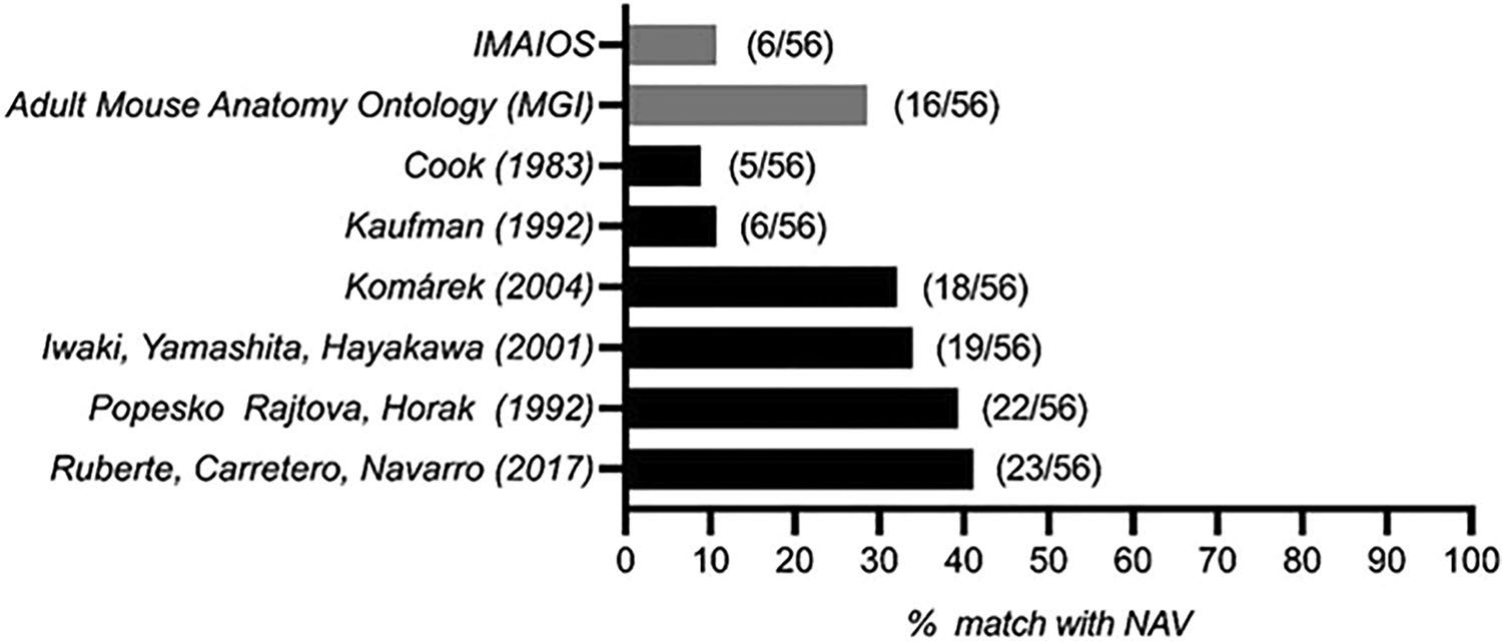
Comparison of mouse forelimb muscles listed in books (black) and databases (grey) with the Nomina Anatomica Veterinaria (NAV)

As an example of the scarce knowledge of muscle anatomy in mice, we only found seven books, two databases, and four articles listing or describing certain of the forelimb muscles. Only less than half of the anatomy books or general “mouse laboratory” books with specific anatomy chapters cited in the previous section contain information about the forelimb muscles, and in many of them no data were available on muscle gross anatomy. When comparing the existing lists of mouse forelimb muscles to the NAV lists of species with five digits, therefore with a similar anatomical complexity, none of the identified resources have even half of the muscles listed in the NAV (Fig. 5). All books surveyed follow the NAV nomenclature, and Komárek (2012) and Ruberte et al (2017) also use the Latin terminology.

Several discrepancies with NAV were found through the analyzed articles. DeLaurier et al. (2008) and Mathewson et al. (2012) describe the dorsoepitrochlearis muscle in the mouse forelimb. This muscle is a typical human muscle variation, not included in TA, which is represented by a muscular or fibromuscular slip detached from the anteroinferior border of the latissimus dorsi muscle to the medial epicondyle of the humerus (Haninec et al. 2009). Similarly, Carry et al. (1993) identified in the mouse forearm the palmaris muscle, which tenses the palmar aponeurosis and flexes the human hand. DeLaurier et al. (2008) name the flexor digitalis superficialis muscle as flexor digitorum sublimis using a former terminology. Finally, (Blackburn et al. 2018) in a superb report defining unique morphogenetic signatures in the mouse muscles, use acromiotrapezius and spinotrapezius to describe respectively the cervical and thoracic parts of trapezius muscle.

## Use of eponyms and other historic anatomical terms

**Fig. 6:**
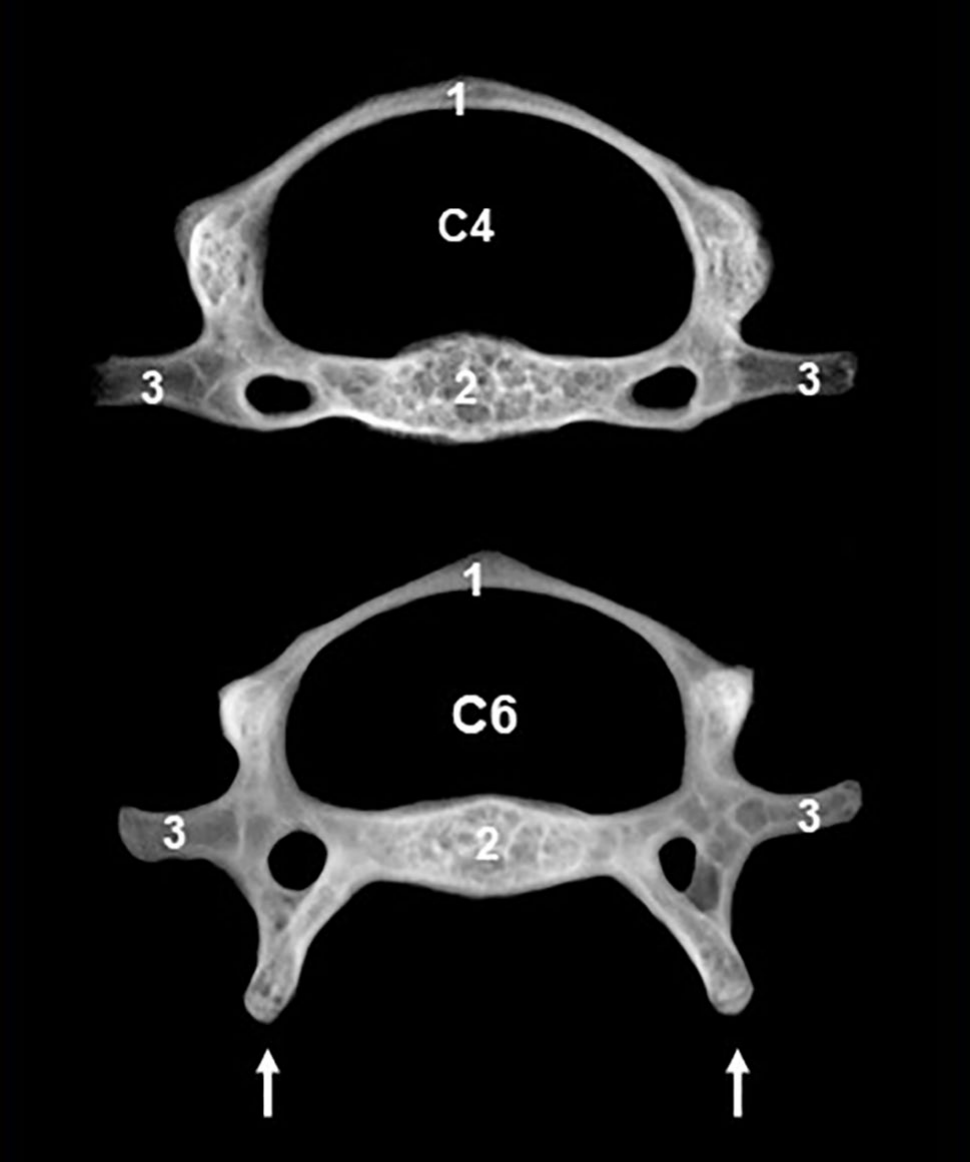
Radiographic appearance of fourth and sixth cervical mouse vertebrae. Note the prominent ventral laminae (arrows) in the sixth vertebra, corresponding to the human carotid tubercles that have also been named as Chassaignac’s tubercles. 1: Vertebral arch; 2: Vertebral body; 3: transverse process

Some anatomical structures received eponyms, terms that incorporate the surname of the people that usually describe them for the first time or studied them (e.g., circle of Willis or follicle of Graff) (Burdan et al. 2016). Eponyms bring color to anatomy, embed medical traditions and culture; however, eponyms lack accuracy, lead of confusion, and hamper scientific discussion in a globalized world (Woywodt and Matteson 2007). Eponyms were officially excluded from anatomical nomenclatures (F.I.P.A.T. 2019; I.C.V.G.A.N. 2017) because an anatomical term should be self-explanatory, which eponyms they are not. In the bibliographic resources analyzed in this commentary, eponyms were not frequently used. An example of eponym is the Wharton’s duct to name the mandibular duct, the route of drainage for the mandibular salivary gland ending at the sublingual caruncle (Kuriki et al. 2011; Jena and Chawla 2021). Another example is the ventral laminae of the sixth cervical vertebra (Fig. 6), that was designated as Chassaignac’s tubercles in mice (Bab et al. 2007), probably because in humans their homologues, the carotid tubercles, where also known as the Chassaignac’s tubercles. They are the longest vertebral anterior tubercles in the human neck and therefore have the potential to compress the carotid arteries against the vertebral body.

Historic anatomical terms not officially accepted by NAV were also used to describe mouse anatomy. One example is the term “innominate (nameless) artery” to identify the brachiocephalic trunk, a branch from the aortic arch that gives rise to the right subclavian and right common carotid arteries supplying the right forelimb, head, and neck. The innominate artery term appeared in the first published mouse anatomy book (Cook 1965), which is still a commonly used reference source for mouse morphological phenotyping. The earliest example of “innominate artery” in the literature is from George Rolleston’s “Forms of Animal Life” (1870). However, several years before the *“*Cyclopaedia of Anatomy and Physiology” (1836), had an entry for brachiocephalic artery. The reason for the historic decision to choose the vague “innominate artery” term is explained in the Mosby’s Medical Dictionary (8th ed.): “the term innominate is traditionally applied to certain anatomic structures, often identified by their descriptive name, such as the brachiocephalic artery”. It is interesting to note that currently the 4th principle of NAV nomenclature points to the contrary: “anatomical terms should have, above all, instructive and descriptive value”.

Both TA and NAV reject the use of eponyms in favor of descriptive and non-honorific terms to ensure clarity and universality in anatomical nomenclature. Regarding this, a relevant issue in current scientific literature is that many journals do not specify which anatomical terminology system should be used in submitted manuscripts. This lack in editorial guidelines contributes to the continued use of eponyms and outdated terms, despite the standardization efforts promoted by TA and NAV. To support accurate integration of phenotypic and genomic data, journals specially in the field of phenogenomics should define and promote the use of standardized anatomical terminology, aligning with recognized authorities such as TA and NAV.

## Do-it-yourself anatomy

The systematic analysis of disease phenotypes in mice, and their correlation with human disease, requires expertise in comparative anatomy (Ince et al. 2008). Anatomy is an important component of the mouse phenotyping pipeline, that should be ideally performed by experts, however, due to insufficient number of qualified specialists to assess these mouse models morphologically, research scientist may perform “do-it-yourself” anatomy resulting in misinterpretation of morphology and phenotype error. For example, the mouse macroscopic preputial glands that humans do not possess have been misinterpreted as lesions (Cardiff et al. 2008). In other cases, protocols developed to dissect specific mouse organs, such as the pancreas (Veite-Schmahl et al. 2017), do not differentiate its main anatomical parts (lobes) or use the corresponding topographic terms (borders and surfaces), which obviously does not allow for an accurate description of the procedure. Nevertheless, some researchers are compelled to perform their own anatomical analyses because their structure of interest is absent from existing terminologies, reports, or anatomical references on the mouse.

**Fig. 7:**
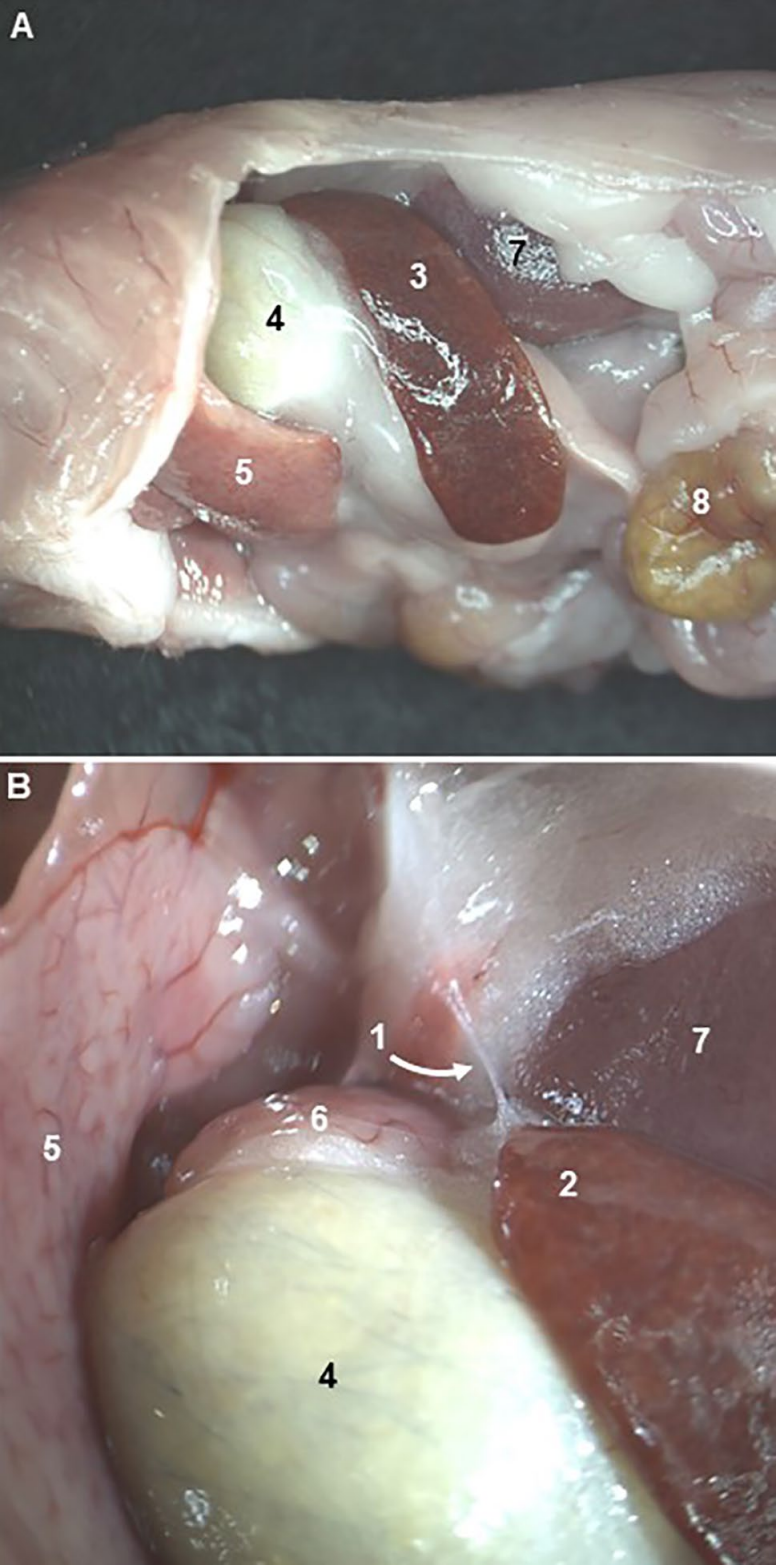
Apical splenic nerve described by Guyot et al. (2019). **A** Left abdominal visceral topography. **B** Apical splenic nerve (1) located at the dorsal extremity (2) of the spleen (3). 4: Stomach; 5: Left lateral lobe (Liver); 6: Papillary process (Liver); 7: Left kidney; 8: Jejunum

An illustrative example is the “apical splenic nerve”, which does not appear in either the NAV or the TA. Guyot et al. (2019) described in the dorsal extremity of the mouse spleen a macroscopic observable nerve referred to as the apical splenic nerve (Fig. 7). Electrical stimulation of this nerve resulted in increased levels of splenic acetylcholine, decreased lipopolysaccharide-induced levels of systemic tumor necrosis factor alpha and mitigated clinical symptoms in a mouse model of rheumatoid arthritis. Later, Cleypool et al. (2022) reviewing the existence of this nerve, confirmed that the apical splenic nerve could be clearly macroscopically identified in mice, and it was running in the phrenicosplenic ligament connecting the diaphragm and cranial pole of the spleen. However, the microscopic evaluation using specific nerve markers showed that this structure did not represent a real nerve, but most likely connective tissue strands. Nevertheless, authors comment that although the apical splenic nerve does not represent a neural structure, its intermediate role in the activation of acetylcholine and anti-inflammatory mechanisms is intriguing and triggers further research.

## Need for new anatomical terms

**Fig. 8:**
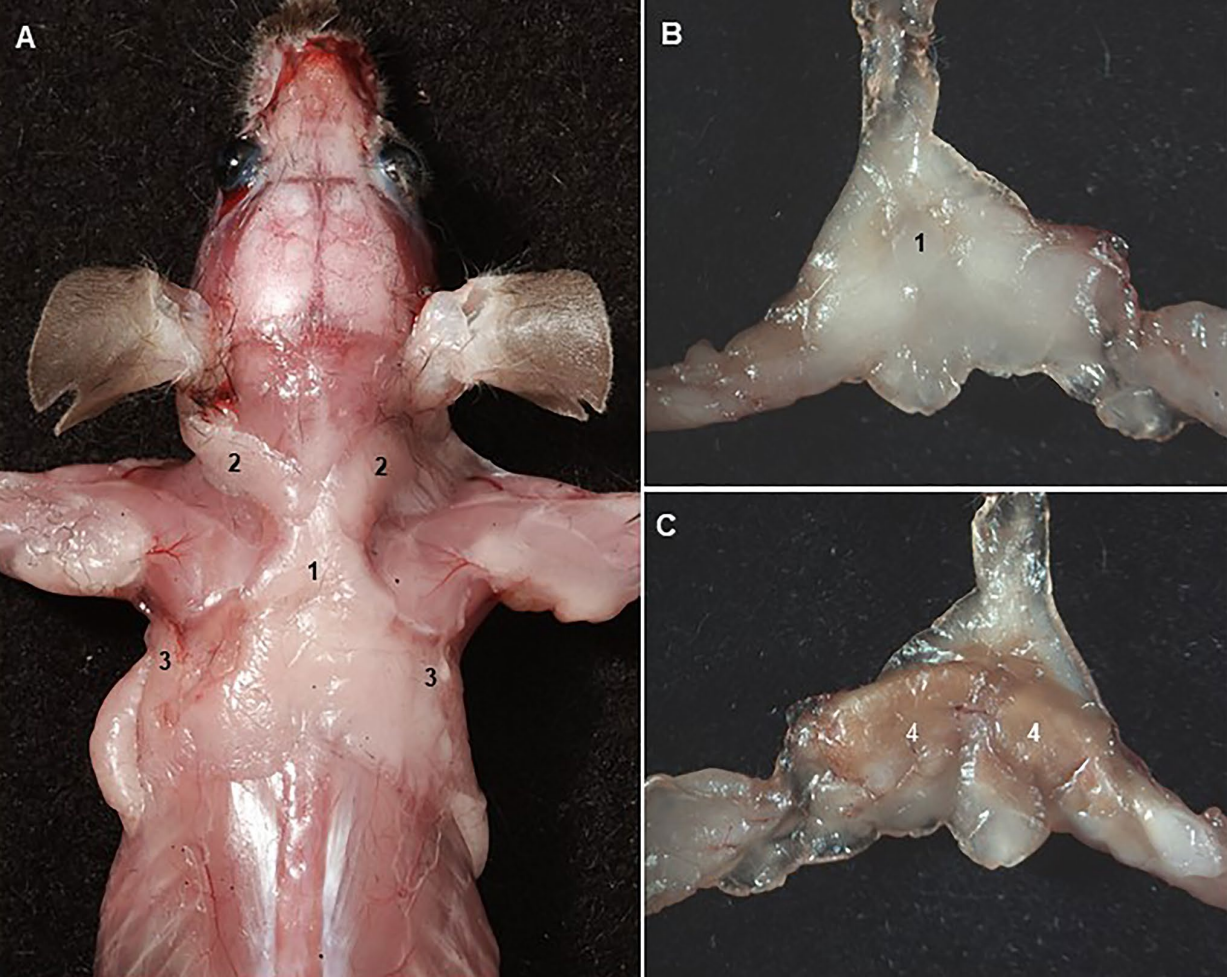
Interscapular fat pad described following the terminology of Zhang et al. (2018). **A** Dorsal vision after removing the skin from the head, forelimbs and trunk. **B, C** dorsal and ventral vision of the isolated interscapular fat pad. 1: Suprascapular; 2: Supraclavicular; 3: Axillary; 4; Classical Brown adipose tissue

The global epidemic of obesity and type 2 diabetes has greatly increased interest in the distribution and function of the adipose organ. Three main types of adipocytes have been described, namely white, beige, and brown fat cells (Scherer 2016). White fat cells are specialized in the storage of energy in the form of triglycerides and thermogenic fat cells, including brown and beige adipocytes, play a critical role in defending against hypothermia, obesity, and diabetes through dissipating chemical energy as heat (Zhang et al. 2018). The adipose organ has been neglected by anatomists. NAV only considers eight fat pads (*corpus adiposi*), including the renal adipose capsule, and none of them are from brown adipose tissue. In adult mice, two main depots of brown adipose tissue are located, one in the area between the scapulae and with several elongated projections abutting toward the cervical region and the axillae (Fig. 8), and other ventral to the vertebral column (Cinti 1999; Zhang et al. 2018).To illustrate the need for new anatomical terms specifically aimed at describing brown fat within the research community, a search in PUBMED showed 7417 results for the words "adipose brown tissue and mice", and 636 results for the words "interscapular fat pad (the most common used unofficial gross anatomy term) and mice”. Therefore, professional anatomists must be more attentive to the needs of the scientific community for the description of anatomical structures not included in the official anatomical nomenclatures.

## Conclusion

In this commentary, we have provided a concise analysis of the current state of anatomical terminology used by the scientific community for the phenotyping of mouse models of human diseases. Through selected examples, we have illustrated a scenario reminiscent of human anatomical nomenclature in the nineteenth century, characterized by inconsistency and lack of coherence. Such terminological fragmentation poses significant challenges for the comparison of newly described mouse phenotypes both among themselves and with human anatomical structures.

The discrepancies identified in the analyzed resources, including the persistence of eponyms and other historically outdated terms, as well as the emergence of "do-it-yourself anatomy" practices, reflect a pressing need to revise the preexisting terminology and introduce new anatomical descriptors not currently included in official nomenclatures, and that are essential for adequately characterizing increasingly sophisticated and detailed disease models.

To address the current limitations in mouse anatomical terminology and its impact on the scientific community, we propose the following coordinated strategic actions:

### Establishment of expert working groups

We propose the creation of expert working groups composed of morphologists and developers of disease models, tasked with reviewing and updating the anatomical knowledge for each mouse organ system. These groups would compile consensus lists of anatomical terms, grounded on ontology databases, such as MA and UBERON, and in current scientific literature. Additionally, these groups would be in charge to ensure alignment with NAV, the internationally accepted standard for quadrupedal species such as the mouse, and TA. The expected impact should promote terminological consistency, improving communication across disciplines, and providing a reliable anatomical framework for accurate mouse phenotyping.

### Development of a Centralized Anatomical Repository

At present, there is no centralized repository for a comprehensive and complete *corpus anatomicum* of the mouse. Given the rapid expansion of anatomical knowledge driven by increasingly detailed phenotypic analyses, it is crucial to integrate and organize this information in a structured and accessible manner. The update and standardization of existing mouse anatomy ontologies, such the MA and EMAPA ontologies (Hayamizu et al. 2013, 2015) used in the GXD resource (Baldarelli et al. 2021) would help to standardize the anatomical corpus, as they are used extensively to annotate phenotypes and gene expression. This would also de facto support anatomical education in the community. We have shown in the example of the right lateral lobe of the liver above, how harmonization and extension of MA and EMAPA would help the development of other ontologies such as UBERON. UBERON is primarily designed to integrate anatomical knowledge across species which is critical for comparing, for example, gene expression patterns or mutant phenotypes in model organisms and humans. It also has a valuable role in semantic standardization, interoperability and knowledge support and education for researchers. Improvement of the granularity and accuracy of mappings between mouse and other species in the cross-species anatomy ontology UBERON should also be supported (Mungall et al. 2012). Enhanced mouse anatomical ontologies would not only help standardize anatomical descriptions but also improve interoperability with other biomedical databases and facilitate advanced data mining.

## Data Availability

No datasets were generated or analysed during the current study.
